# Wolves alter the trajectory of forests by shaping the central place foraging behaviour of an ecosystem engineer

**DOI:** 10.1098/rspb.2023.1377

**Published:** 2023-11-08

**Authors:** Thomas D. Gable, Sean M. Johnson-Bice, Austin T. Homkes, John Fieberg, Joseph K. Bump

**Affiliations:** ^1^ Department of Fisheries, Wildlife, and Conservation Biology, University of Minnesota, 2003 Upper Buford Circles, St Paul, MN 55108, USA; ^2^ Department of Biological Sciences, University of Manitoba, 50 Sifton Road, Winnipeg, Manitoba, Canada, R3T 2N2

**Keywords:** wolves, non-consumptive effects, predation, central place foraging, ecosystem engineer, ambush behaviour

## Abstract

Predators can directly and indirectly alter the foraging behaviour of prey through direct predation and the risk of predation, and in doing so, initiate indirect effects that influence myriad species and ecological processes. We describe how wolves indirectly alter the trajectory of forests by constraining the distance that beavers, a central place forager and prolific ecosystem engineer, forage from water. Specifically, we demonstrate that wolves wait in ambush and kill beavers on longer feeding trails than would be expected based on the spatio-temporal availability of beavers. This pattern is driven by temporal dynamics of beaver foraging: beavers make more foraging trips and spend more time on land per trip on longer feeding trails that extend farther from water. As a result, beavers are more vulnerable on longer feeding trails than shorter ones. Wolf predation appears to be a selective evolutionary pressure propelled by consumptive and non-consumptive mechanisms that constrain the distance from water beavers forage, which in turn limits the area of forest around wetlands, lakes and rivers beavers alter through foraging. Thus, wolves appear intricately linked to boreal forest dynamics by shaping beaver foraging behaviour, a form of natural disturbance that alters the successional and ecological states of forests.

## 1. Introduction

Predators directly and indirectly alter the foraging behaviour of their prey through direct predation (consumptive effects) or the fear of predation (non-consumptive effects) [1–3]. Predation, or the risk thereof, can affect when prey forage [4,5], where prey forage [6,7], how long prey forage in particular areas [8,9], and the intensity of prey foraging [10]. Furthermore, predation can shape prey foraging behaviour by reducing or increasing fitness costs associated with particular foraging strategies [1,11]. By altering the foraging behaviour of their prey, predators can indirectly influence the growth and regeneration of lower trophic levels that prey species consume [12–14]. In doing so, predators can initiate ecosystem-level effects that influence myriad species and ecological processes [15,16].

Central place foragers have unique foraging constraints and decisions because all foraging activity radiates out from a central location [17,18]. Much work has been done to understand how central place foragers make movement decisions [19]; in particular, how central place foragers balance the energetic costs and rewards of foraging at greater distances from the central place with the predation risk of doing so [20–22]. Implicit in this previous work is that central place foragers are at a higher risk of encountering and being killed by predators at greater distances from the central place [23]. However, there is little empirical evidence to demonstrate that prey encounter, and are killed more frequently by, predators farther from the central place. Instead, most studies have examined how central place foraging prey respond to proxies of predation risk (e.g. predator scent, sound, facsimiles or other cues) at varying distances from the central place to indirectly evaluate how predation shapes foraging decisions [22,24]. Researchers assume the proxy sufficiently represents predation risk to prey, and thus the prey's response indicates the extent to which predators alter foraging behaviour [7]. However, studies using these approaches do not necessarily simulate predation risk as experienced by prey [23,25], nor do they account for both the consumptive and non-consumptive aspects of predation [2].

Empirical evidence of how predators hunt and kill central place foraging prey in relation to distance from the central location would be beneficial for assessing how predators can alter central place foraging behaviour [7]. Understanding this relationship would be particularly insightful because central place foragers can alter localized ecological dynamics around central places [26,27], and ultimately affect larger ecosystem-level processes [28–30]. For example, western fence lizards (*Sceloporus occidentalis*) altered plant biomass and vegetation structure by reducing the abundance of grasshoppers—their primary prey—around their central place [28]. Thus, predators could, by constraining or altering the foraging behaviour of central place foraging prey, indirectly alter larger ecological processes [31].

The widespread distribution of beavers (*Castor* spp.) and the relative ease of studying their foraging decisions (beavers forage along conspicuous ‘feeding trails’ and leave visible stumps after cutting trees) has led to extensive research on central place foraging behaviour using beavers as a model species [20,24,32]. In particular, much empirical and experimental work has examined the extent to which beavers balance energy maximization and predation risk while foraging [20,33–35]. Such work has shown that while beavers generally follow an energy maximization strategy, their foraging strategies are modified by environmental or biological factors (i.e. predation risk) that constrain and alter foraging behaviour [32]. These findings comport with numerous studies that have concluded, based on various indirect evidence, that beavers preferentially forage closer to the safety of water to minimize predation risk [36–39]. All of this indicates that predators shape beaver foraging behaviour through non-consumptive mechanisms (i.e. beavers reduce foraging distance in response to predation risk [31]) or via consumptive mechanisms where predators disproportionately remove individuals who forage farther from water. However, there has been no direct evidence that predators hunt or kill beavers more frequently farther from water [23].

Wolves (*Canis lupus*) and beavers co-occur across most boreal ecosystems in North America and Eurasia [40]. Wolves are the primary predator of beavers wherever the two species co-occur, and beavers are important seasonal prey for wolves [40]. Although wolves are primarily cursorial predators, they often use ambush strategies to hunt and kill beavers [41,42]. Still, a substantial proportion of predation is the result of opportunistic encounters [23,43]. Because wolves are apex predators [44] and beavers are ecosystem engineers [45], wolf predation on beavers can have outsized ecological effects. For example, by killing dispersing beavers, wolves alter the creation and recolonization of wetlands, and in turn, alter all of the ecological effects associated with beaver-created wetlands [46]. Logically, wolf predation could also have indirect ecological effects by shaping beaver foraging behaviour.

**Figure 1.**
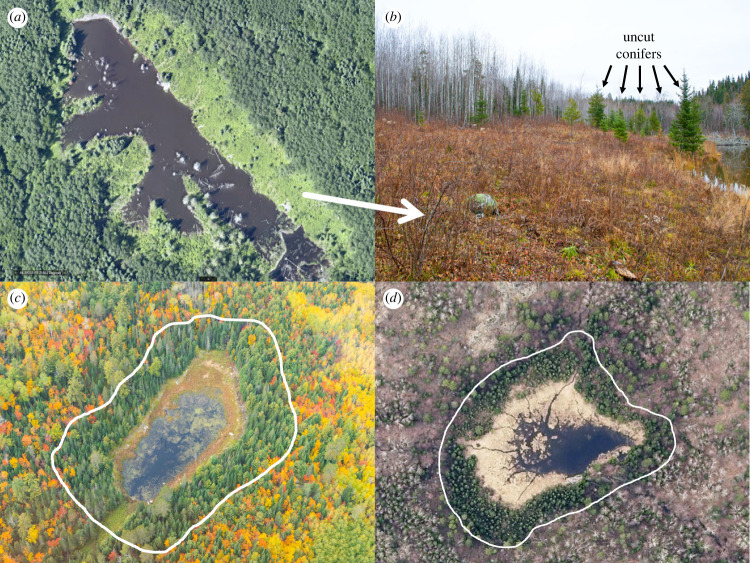
Beavers alter the structure and composition of forests around their ponds by selectively cutting preferred deciduous tree species in the Greater Voyageurs Ecosystem, MN, USA. Their selective foraging can convert forests around wetlands and waterways to early successional states such as in (*a*) and (*b*), where beavers removed almost every aspen tree within 20–30 m of the pond. The only trees that beavers did not cut were conifers (*b*). Over time, the forests around beaver ponds become dominated by conifers (*c,d*) or less preferred deciduous species. This conversion to conifer-dominated forests often results in a conifer ring or halo (white line in (*c*) and (*d*)) around shorelines that is visible from aerial imagery. Photo credit for (*a*) and (*d*): St. Louis County, MN; photo credit for (*b*) and (*c*): Tom Gable.

Beavers, by selectively cutting and felling trees, are agents of natural disturbance that alter and shift the trajectory of forests around wetlands and shorelines (figure 1) [45,47,48]. Beavers primarily alter forest composition and structure by (1) resetting forest succession by creating gaps in the canopy which allow early successional, shade-intolerant species (e.g. aspen) to thrive (figure 1*a,b*) [47], (2) shifting forest communities toward deciduous stands of tree species less preferred by beavers (e.g. black ash and alder) [49], and/or (3) converting deciduous or mixed forests to conifer-dominated forests by selectively removing deciduous trees (figure 1*c,d*) [31,50,51]. In the latter instance, wetlands or waterways become surrounded by a well-defined narrow ring, or halo, of conifer forest (figure 1) [31,51]. Notably, the area of forest beavers alter in an ecosystem is directly related to the distance from water that beavers forage; foraging at increased distances from water increases the area of forest altered and *vice versa*. Therefore, any factor that alters beaver foraging behaviour would undoubtedly have reverberating effects on forests themselves. Peterson *et al*. [31] posited, based on aerial imagery and beaver foraging patterns, that wolves altered forests on Isle Royale National Park by limiting the distance from water beavers forage, although this idea has yet to be tested.

Herein, we describe and demonstrate how wolf predation is a selective pressure that shapes the foraging behaviour of beavers via consumptive and non-consumptive mechanisms in the Greater Voyageurs Ecosystem (GVE), Minnesota. By altering beaver foraging behaviour, wolves invariably alter the ecological trajectory of forests around wetlands and beaver-occupied water sources (e.g. lakes, rivers).

## 2. Study area

Our study was conducted as part of the Voyageurs Wolf Project, which studies the ecology of wolves and their prey in and around Voyageurs National Park, MN, USA, an area we refer to as the GVE. The GVE is typical of a southern boreal ecosystem situated in the Laurentian Mixed Forest Province. The landscape is typified by dense forests (deciduous, coniferous and mixed) and abundant lakes, bogs, and wetlands interspersed with outcrops and rocky ridges from glacial activity. The GVE has sustained dense wolf (average density of 58 wolves per 1000 km^2^ [52]) and beaver populations (>0.47–2.0 colonies per km^2^ [53,54]) for more than 30 years. Beavers are important seasonal prey for wolves in the GVE with beaver constituting up to 42% of wolf pack diets from April to October (the ice-free season) when beavers are vulnerable to predation [55]. For more information on the GVE, see Gable *et al*. [46].

## 3. Methods

### (a) Wolf predation behaviour from searching clusters of GPS locations

During 2015–2022, we captured wolves using foot-hold traps and cable restraints and fitted them with GPS collars. We then visited clusters of GPS locations during April–October to identify predation events and ambush locations (Institutional Animal Care and Use Committee: MWR_VOYA_WINDELS_WOLF and UMN protocol no. 2207-40241A). We considered a cluster to be ≥2 consecutive locations ≥20 min apart and within a 200 m radius of one another based on previous work indicating these cluster criteria are sufficient to study wolf predation on beavers [23,46]. On average, we searched clusters within 5.8 days after the cluster occurred and each cluster was searched once.

When at GPS clusters, we searched the area systematically for evidence of a predation event. Such evidence included tufts of fur, stomach contents, skull/lower mandible, bones and castor glands in an area of disturbance or trampled vegetation [41]. When we located wolf-killed beavers, we noted the habitat feature (e.g. feeding trail, dam) at which the beaver was killed [23]. Additional information on GPS-cluster search methods can be found in previous studies by Gable *et al*. [23,41]. If beavers were killed at feeding trails, we measured the feeding trail length (distance from water to the end of the trail) to the nearest metre by walking the contour of the trail [23]. In other words, trail length is not the straight-line distance from the end of the trail to water but rather the distance the beaver would have to move along the trail from water to reach forage at the end of the trail (i.e. a beaver's travel path). Nonetheless, beaver feeding trails are relatively straight trails that extend perpendicularly from water to where beavers are actively cutting trees. Beavers almost always forage at the end of feeding trails (i.e. they do not create trails that are any longer than needed to secure forage), and trails only become longer as beavers venture further inland to cut additional trees.

We also documented instances where wolves waited in ambush for beavers but did not make a kill (i.e. ‘ambush attempts’). We defined ambush attempts to be ≥2 consecutive locations <25 m apart, of which 50% had to be ≤15 m from fresh beaver activity (e.g. fresh cuttings and mud on scent mound [23,41]). In other words, ambush attempts were a tight cluster of wolf locations near recent beaver activity but where no kill was found. When ambush attempts were found, we recorded the beaver habitat feature (e.g. feeding trail, beaver dam, lodge; [23]) at which wolves waited. If ambush attempts were along active feeding trails (those with fresh or recent beaver activity), we recorded the length of the feeding trail to the nearest metre [23]. However, we did not record data on feeding trail length at ambush attempts before 2018.

### (b) Temporal patterns of beaver foraging using remote cameras

To understand and assess temporal patterns of beaver foraging behaviour, we deployed remote cameras on feeding trails in 2017 and 2018. Detailed methods are available in electronic supplementary material, A. Briefly, we deployed cameras on three active feeding trails at individual beaver colonies for two weeks in the spring, summer, and fall to assess seasonal changes in foraging behaviour. We assumed active trails were those with fresh cuttings, trampled vegetation and other signs indicative of recent beaver use. We recorded the length of feeding trails that cameras were deployed on. Using remote camera photographs, we calculated the number of foraging trips beavers made on trails and the duration of these foraging trips.

### (c) Assessing spatial patterns of beaver foraging from feeding trails

We collected additional field data on beaver foraging for each colony at the end of each camera deployment period for each of the three seasons to assess seasonal changes in spatial patterns of beaver foraging. Specifically, we measured the length (nearest metre) of all new active feeding trails as well as the total number of active feeding trails used by a colony. As described above, we considered active trails those that had signs of recent beaver use (e.g. fresh cuttings, trampled vegetation). At pond colonies, we recorded all active trails around the entire perimeter of the primary pond and any secondary ponds that the colony was using. Based on previous work on the movement patterns and foraging behaviour of beavers in the GVE [56,57], we recorded all active trails at lake colonies that were within a 400 m radius of the colony's lodge. These feeding trails (hereafter referred to as ‘reference trails’) should be a representative sample of the feeding trails that beavers use for foraging and should represent the distance from water that beavers generally forage.

### (d) Analyses

**Figure 2.**
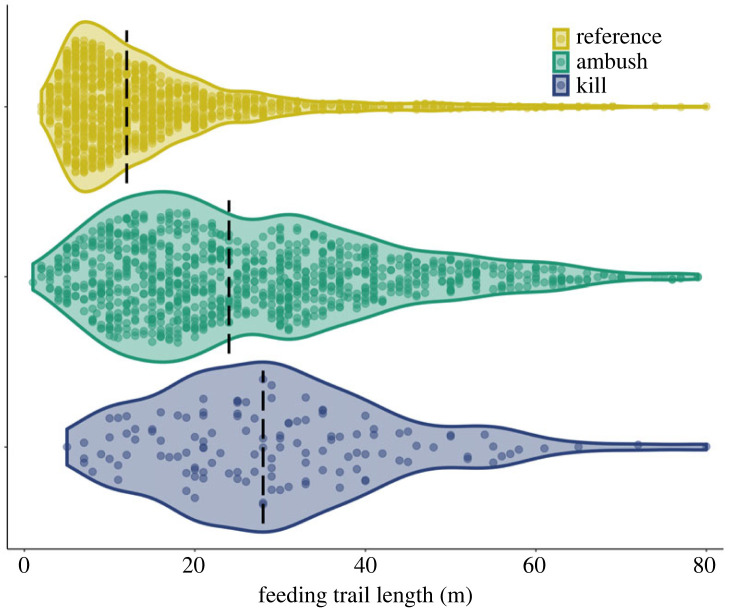
The length of feeding trails that beavers foraged along (‘reference’, *n* = 1111), that wolves waited in ambush along (‘ambush', *n* = 949) and that wolves killed beavers along (‘kill', *n* = 128) the Greater Voyageurs Ecosystem, MN, USA. Each plot shows the distribution of the raw data collected on each trail type and the individual circles within each violin plot represent individual feeding trails. The dashed black line represents the median value of the raw data.

We estimated the typical length of reference trails (determined by assessing spatial patterns of beaver foraging) using linear mixed-effects models. Similarly, we used linear mixed-effects models to estimate the typical length of trails where wolves waited in ambush (ambush trails) and where they killed beavers (kill trails). Because trail lengths were right skewed (figure 2), log transformations were used to better meet the assumptions of the models (e.g. normally distributed errors with constant within-cluster variance). We considered using logistic regression for our analysis but doing so would have prevented us from capturing valuable aspects of the study design; in particular, repeated measures at the beaver-colony level (for reference trails) and at the wolf-level (for ambush and kill trails). Specifically, we fitted separate models to these two data sets (reference beaver feeding trails and wolf ambush/kill trails), including random effects associated with ‘colony ID’ in the former model and ‘wolf ID’ in the latter. For reference feeding trails, we modelled how the log of trail length varied by season (spring/summer/fall) and colony type (pond/lake) with a random intercept included for each colony ID and a set of random slopes to allow seasonal effects to vary by colony. For kill and ambush trails, we modelled how the log of trail length varied based on the interaction of trail type (kill/ambush) and season (spring/summer/fall) with a random intercept for each wolf and a set of random slopes to allow seasonal effects to vary by wolf. We used coefficients and their standard errors from the two different models (reference versus kill/ambush) to calculate *z*-scores and *p*-values to assess if there were discernible differences in the median length of reference versus ambush trails and reference versus kill trails across seasons (e.g. we compared ambush trails in fall to reference trails in fall and kill trails in fall to reference trails in fall).

We used a negative binomial mixed-effect model to assess how the number of foraging trips on a trail varied as a function of trail length, season and the number of trails used by a colony. We scaled and centred trail length and number of trails used by a colony to have mean = 0 and s.d. = 1. We used the log of camera deployment duration (in days) as an offset to account for variable deployment period (13–17 days). We included a random intercept for colony ID and attempted to include a set of random slopes to allow seasonal effects to vary by colony, but the model would not converge. Thus, we used the simplified random intercept only model for inference.

To assess factors influencing foraging trip duration, we used a linear mixed-effect model to examine how the log of foraging trip duration varied as a function of feeding trail length, season and number of trails used by a colony. We included a random intercept for colony ID and a set of random slopes to allow seasonal effects to vary by colony.

We then estimated a cumulative distribution function (CDF) to represent the distribution of time spent by beavers on trails of different trail lengths. Importantly, this approach allowed us to estimate the cumulative time beavers spend foraging as distance from water increases. To do so, we: (1) determined a representative set of trail lengths by resampling lodges with replacement from our reference trail data set (we included all trails associated with those lodges); (2) generated predictions for these trails, both for the number of trips and time spent per trip, using generalized linear mixed-effect models parameterized using data from the remote cameras; and (3) multiplied the number of trips by the time spent per trip to get a distribution of the time spent foraging on trails of different trail lengths. To capture uncertainty in the CDF representing the time beavers spent foraging on trails of different trail lengths, we repeated this process 1000 times, incorporating uncertainly in the parameters of the generalized linear mixed-effect models using a parametric bootstrap. We then estimated CDFs representing the relative frequencies of wolf ambush and kill events as a function of trail length. We used a cluster-level bootstrap [58], resampling wolf IDs with replacement, to represent uncertainty in these estimated CDFs. All analyses were conducted using the R programming language and the mixed-effects modelling was done with the *lme4* package [59].

### (e) Estimating the indirect effects of wolves on forest structure and composition

We sought to estimate how forest area affected by beaver foraging at a landscape-scale changes as a function of how far beavers forage from water. In brief, we digitized all beaver-created ponds—occupied or abandoned—in the GVE using high-resolution aerial imagery [49,60,61] and long-term aerial beaver survey data [46,62]. Then, we used the multiple ring buffer tool in ArcGIS Pro 2.8 to create ‘forage buffers’ around the outside of each pond complex or lodge (for lake/river colonies) from 1 to 30 m in 1 m increments. We then summed the area of all forage buffers at 1 m increments (1 m, 2 m, … 30 m) to describe how forest area available to beaver foraging in the GVE increased with increasing distance from water. We then fitted a quadratic equation (*R*^2^ = 0.999) to these results that allowed us to calculate the forest area available to beaver foraging at any given distance from water. For a more detailed description of these methods, see electronic supplementary material, B and C.

We then used this information to estimate the forest area wolves alter or influence by constraining beaver foraging (see electronic supplementary material, C). We first determined the amount of forest in the GVE that wolves would indirectly alter if they constrained beaver foraging by a single metre. We then attempted to estimate the extent to which we think wolves might actually constrain beaver foraging, and indirectly alter forests. To do so, we assumed the median distance beavers would forage in the absence of wolves would be the same as the predicted median length of kill trails. In other words, we assumed the forest area between predicted median length of reference and kill trails, based on our mixed-effects modelling approach as described above, was generally indicative of the extent to which wolf predation constrains beaver foraging (i.e. reduces the area beavers predominantly forage in). Importantly, our objective in using this approach was to understand the relative magnitude of the indirect effect of wolf predation on forests (*sensu* [46]). Estimates generated with this approach are coarse but helpful for thinking about how wolf predation might alter ecological processes at the landscape scale. We calculated predicted median length of reference and kill trail values using the ‘ggemmeans()’ function in the *ggeffects* package in R [63]. Although we modelled trail length (as described above) on the log scale (i.e. log[trail length]), we obtained predicted values on the original scale (m). Thus, the predicted values represent the median trail length of the median individual (wolf or colony) in our sample.

## 4. Results

We searched 27 741 clusters of GPS locations from 51 wolves during 2015–2022. In doing so, we identified 543 wolf-killed beavers and 1909 instances where wolves attempted to ambush beavers. Of the 543 wolf-killed beavers, 135 (25%) were killed on feeding trails and we recorded the length of feeding trails at 128 of these kills. Of the 1909 ambushing attempts, 949 (50%) were at feeding trails. All other kills and ambush attempts occurred at other beaver features including beaver dams, scent-mounds, feeding canals and lodges.

### (a) Beaver foraging and wolf predation based on GPS-clusters and feeding trails

**Figure 3.**
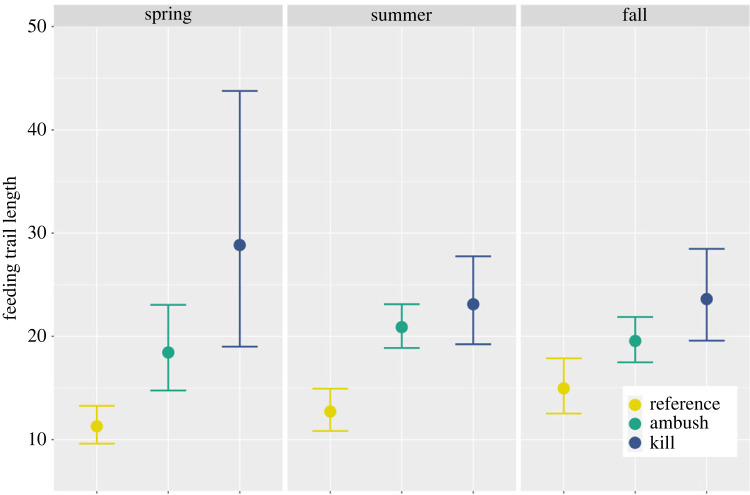
The relationship between feeding trail length of beavers, trail type (reference, ambush or kill trails), and season in the Greater Voyageurs Ecosystem, MN, USA based on linear mixed-effect models. Reference trails (*n* = 1111) were a representative sample of feeding trails that beavers used for foraging, ambush trails (*n* = 949) were feeding trails where wolves waited in ambush for beavers, and kill trails (*n* = 128) were feeding trails where wolves killed beavers. Point estimates and 95% confidence intervals were obtained by setting all random effects to 0 and then exponentiating the estimated log means. Thus, these point estimates represent estimated median trail lengths for a typical colony or wolf with all random effects set equal to 0 [64]. The large confidence interval for kill trails in spring is due to small sample size (*n* = 10).

To assess reference feeding trail length in the GVE, we measured 1111 beaver feeding trails from 36 beaver colonies during 2017–2018 (figure 2). Spring feeding trails were on average shorter than fall trails (*ß*_spring_ = −0.28, 95% confidence interval [CI] = −0.49 to −0.09) but we did not detect a difference between spring and summer trail length or summer and fall trail length (figure 3). We did not detect a difference in feeding trail length between beaver colonies living in lakes or ponds (*ß* = −0.08, 95% CI = −0.34 to 0.17). Ambush (*n* = 949) and kill trails (*n* = 128) were, on average, longer than reference feeding trails regardless of season (*p* < 0.05; figures 2 and 3), but we did not detect a difference in length between ambush and kill trails (figure 3).

### (b) Temporal patterns of beaver foraging using remote cameras

**Figure 4.**
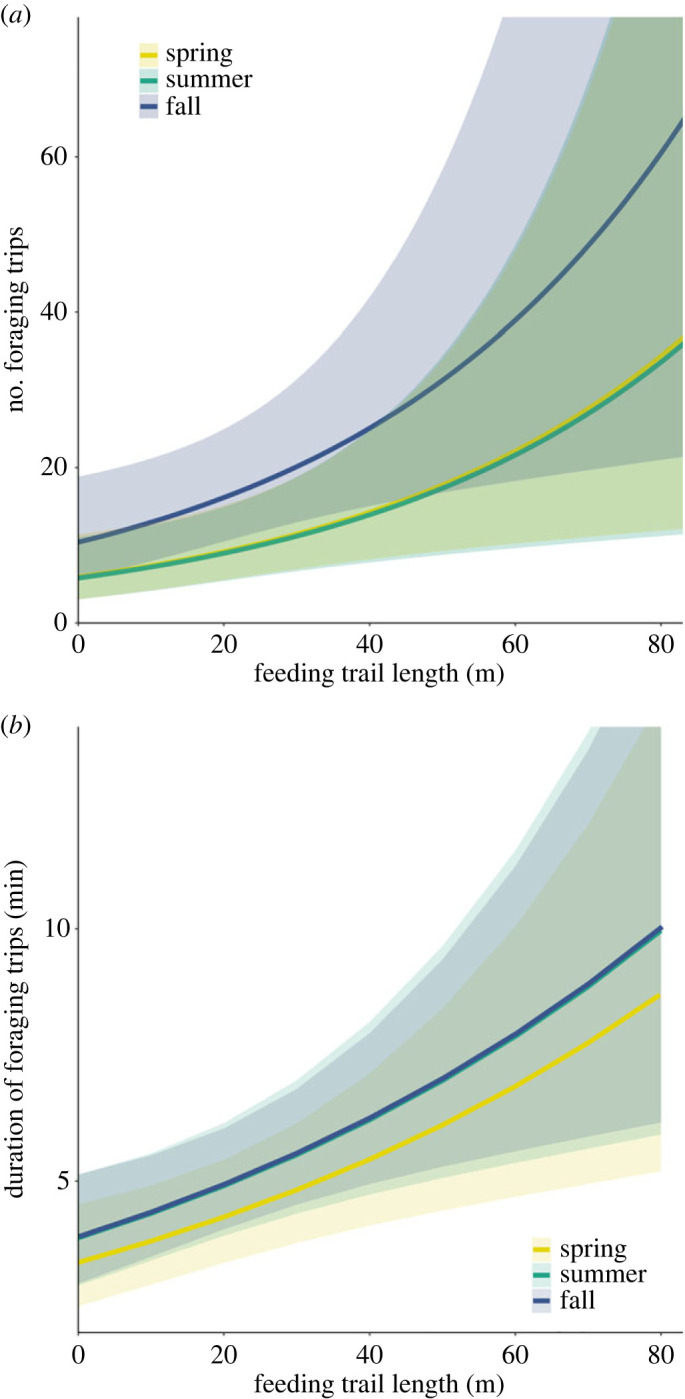
The relationship between feeding trail length and the number (*a*) and duration (*b*) of foraging trips made by beavers on feeding trails in the Greater Voyageurs Ecosystem, MN, USA. The lines and shaded 95% confidence intervals for each season were obtained using a negative binomial mixed-effect model (*a*) and linear mixed-effect model (*b*), fixing all other variables at their mean values.

We placed remote cameras on 201 feeding trails across 35 colonies during 2017–2018. Our cameras took 145 295 pictures which yielded 4705 terrestrial beaver events. We recorded beavers exiting and entering the water (i.e. round-trip foraging events) in 2113 events that occurred at 118 feeding trails. The number (*ß* = 0.35; 95% CI = 0.08 to 0.63) and duration (*ß* = 0.010; 95% CI = 0.002 to 0.018) of foraging events on feeding trails increased with feeding trail length (figure 4). After adjusting for trail length, the duration of foraging events did not vary across seasons, but the number of foraging events was higher on feeding trails in fall than in spring or summer (*ß*_spring_ = −0.57, 95% CI = −1.13 to 0.006, *p* = 0.051; and *ß*_summer_ = −0.59, 95% CI = −1.12 to −0.05, *p* = 0.03; figure 4). We did not detect an association between the number of active feeding trails used by a colony and the duration of foraging events (*ß* = −0.006; 95% CI = −0.014 to 0.0007) or the number of foraging events on feeding trails (*ß* = −0.10; 95% CI = −0.39 to 0.25).

**Figure 5.**
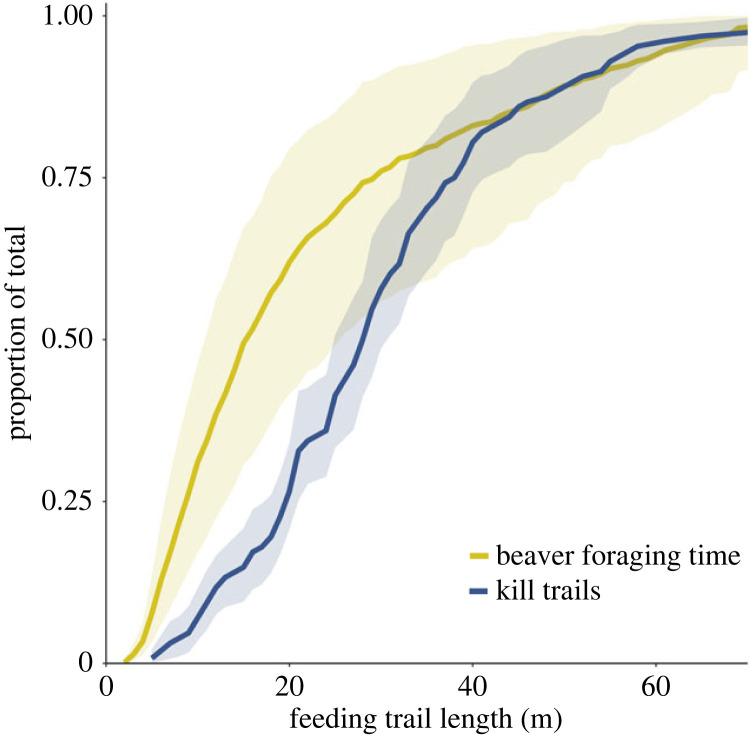
The cumulative proportion of time beavers spent foraging on feeding trails of length ≤*x* (yellow line) and the cumulative proportion of kill trails (blue line) that were of length ≤*x*. We would expect the curves to be similar if wolves were killing beavers in proportion to beavers' spatio-temporal availability. However, the ‘kill trails’ curve is significantly different from the beaver foraging curve, indicating that wolves killed beavers at disproportionately longer trails than would be expected based on the spatio-temporal availability of foraging beavers.

Although the expected number of foraging trips and expected time spent per foraging trip increased with feeding trail length, beavers cumulatively spent most of their time foraging on shorter trails (figure 5). Indeed, beavers spent an estimated 50% (95% CI: 32–69%) of their time foraging on trails less than ≤15 m and 75% of their time foraging on trails ≤28 m. By contrast, only 15% and 24% of kills and ambushes, respectively, occurred on trails ≤15 m long, and 50% and 57% occurred on trails ≤28 m long. This contrast is apparent in the CDF curves, which indicate wolves disproportionately killed and ambushed beavers at longer trails than beavers chose to use most often (figure 5).

### (c) Estimating the indirect effect of wolves on forests

**Figure 6.**
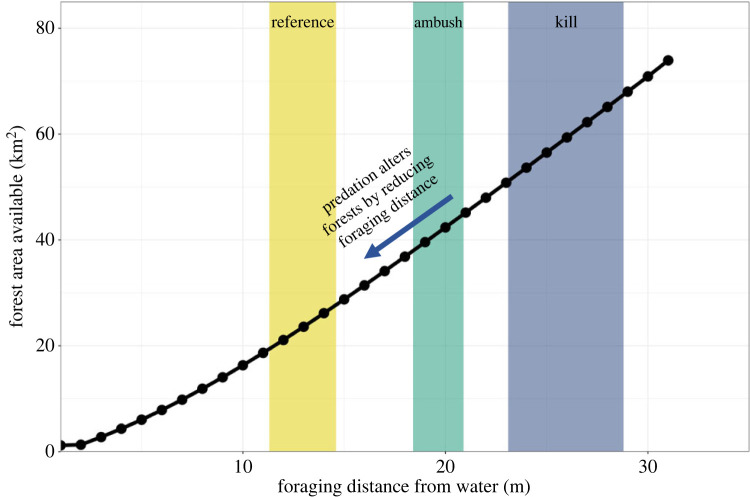
How forest area available to beaver foraging in the Greater Voyageurs Ecosystem, MN, USA increases as beavers forage at greater distances from water. The yellow bar represents the range of median feeding trail lengths across seasons (spring–fall) for a typical beaver colony with random effects set to 0 [64]. Reference feeding trails were a representative sample of feeding trails that beavers used for foraging and should represent the distance beavers generally forage. The green and blue bars represent the range of median ambush (feeding trails where wolves waited in ambush for beavers) and kill trail lengths (feeding trails where wolves killed beavers) across seasons (spring–fall) for a typical wolf with random effects set to 0. Our work indicates that wolf predation, or the risk thereof, shifts the density of beaver foraging closer to water and reduces the amount of forest beavers alter through their selective foraging.

We digitized 7175 beaver-created wetlands using aerial imagery and 1317 beaver lodges along lakeshores and rivers that were identified via aerial surveys. We then assessed how the total amount of forest area available to beavers for foraging (i.e. the foraging buffers described earlier) increased with distance from water (figure 6). Using these calculations, we estimate that for every metre that wolves constrain beaver foraging, they indirectly influence or alter—depending on distance from water—1.3–2.9 km^2^ of forest in the GVE (figure 6).

**Figure 7.**
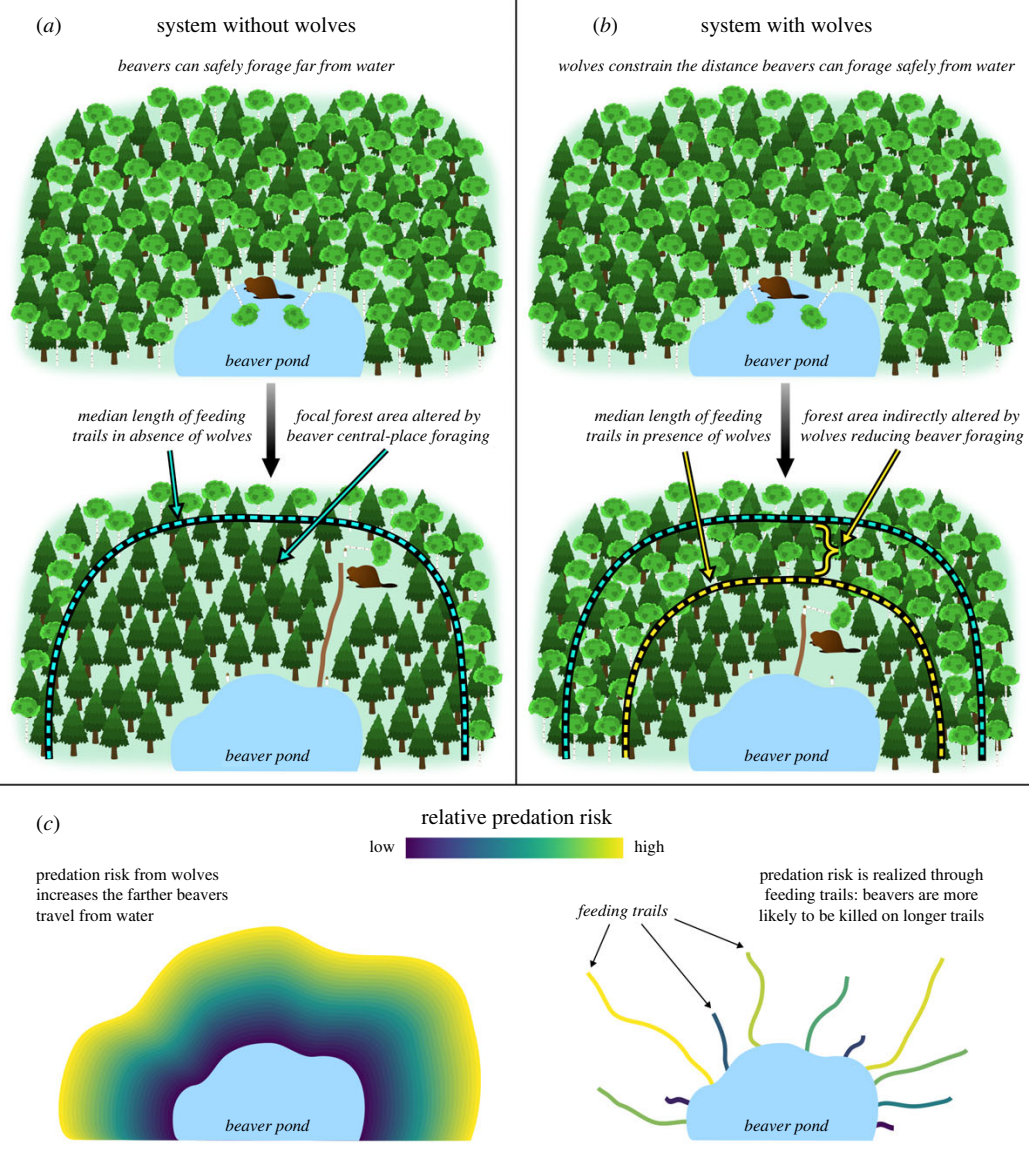
Conceptual diagram depicting how wolves indirectly alter the trajectory of forests by constraining the distance beavers forage from the safety of water. In a system without wolves (*a*), beavers can safely forage farther from water than they would be able to in a system with wolves (*b*). Consequently, the median length of feeding trails in a system with wolves (dashed yellow/black line in (*b*)) is less than the median length of feeding trails in a system without wolves (dashed turquoise/black line in (*a*,*b*)). In this study, we assumed the focal area most affected by beaver central-place foraging was restricted to the area of forest between the water's edge and the median length of a typical beaver trail (11.3–14.9 m; yellow/black line). We assumed the median length of feeding trails where wolves killed beavers (23.1–28.8 m) represented the upper limit of potential median feeding trail lengths in a system without wolves (turquoise/black line). The area of forest between these two median feeding trail lengths represent the potential area of forest indirectly altered by wolves through their effect on beaver foraging (*b*). Panel (*c*) represents our hypothesis on how these foraging patterns are formed. Predation risk increases the farther beavers travel from water (*left*), but this risk is realized through beaver feeding trails (*right*): since most terrestrial foraging by beavers is restricted to feeding trails, predation risk is directly related to feeding trail length, such that beavers are more likely to be killed while traveling on longer feeding trails that extend farther from the water's edge.

Predicted median length of reference trails for the typical beaver colony was 11.3–14.9, depending on season, whereas predicted median length of kill trails for the typical wolf, depending on season, was 23.1–28.8 (figure 3). These values indicate wolves may constrain beaver foraging by up to 8.7–17.5 m (estimated difference in median trail length between kill and reference trails is 8.7 m in fall and 17.5 m in spring; figure 3) and may have reduced the area beavers predominantly forage in by as much as 43–69% (figure 7). As such, we estimate wolves may be indirectly influencing the structure and composition of roughly 23–47 km^2^ of forest by altering beaver foraging behaviour (figure 6). Put differently, we estimate wolves may indirectly influence or alter up to 1.4–2.9% of the forest in the GVE (GVE landmass = 1617 km^2^).

## 5. Discussion

We present direct evidence that wolves, through ambush behaviour and direct killing, constrain beaver foraging closer to the safety of water. Wolves killed and ambushed beavers at substantially longer feeding trails than would be expected based on the spatio-temporal availability of foraging beavers—a pattern that is probably driven by the fact that beavers make more and longer foraging trips on longer feeding trails (figures 3 and 4). These patterns indicate wolf predation is likely a selective evolutionary pressure propelled by both consumptive and non-consumptive mechanisms that shape beaver foraging behaviour. Because beavers are a source of natural disturbance that alters the structure and composition of forests through their tree-cutting behaviour, we argue wolves indirectly alter the trajectory of forests around wetlands and other water features (e.g. lakes, rivers) by constraining beaver foraging behaviour.

### (a) Predation as a selective pressure

Wolf predation appears to be a strong selective pressure on beaver foraging behaviour. The predicted median length of ‘kill trails’ was 58–154% longer, depending on season, than the predicted median length of reference feeding trails (figure 3). This pattern strongly suggests that predation risk for beavers is greater on longer trails and that wolves, and likely other predators, play a key evolutionary role in shaping beaver foraging by disproportionately removing individuals that forage farther from water. Wolf predation could be a particularly strong selective pressure on foraging behaviour because older beavers—typically breeding adults—often forage on land more frequently than younger beavers [65–68]. Thus, wolves may kill breeding individuals that forage farther from water more often and, more importantly, kill breeding individuals that forage closer to water less often. We speculate that over time, breeding beavers that forage closer to water might have higher fitness and produce offspring with similar foraging behaviours.

Beavers are more vulnerable to predation on longer trails because the duration of foraging trips increases with trail length as does the frequency of foraging trips (figure 4). The reasons for this are probably two-fold: (1) the time needed to travel to and then transport cut trees back to water increases with distance from water [20], and (2) beavers in the GVE, similar to many other areas (e.g. [49,69]), selectively cut larger diameter trees as distance from water increases [70]. Although larger diameter trees require more time and trips to cut and transport back to water relative to smaller trees, they yield substantially more food [20,33,35]. As beavers forage further inland, they appear to cut larger diameter trees to ensure that the reward of foraging farther from the safety of water outweighs the increased risk of predation [35,71]. However, beavers in the GVE clearly prefer to forage in close proximity to water when possible, as 50% of the time beavers spent foraging occurred on trails ≤15 m long and 76% on trails ≤30 m long (figures 2 and 5).

### (b) Ambushing behaviour as a non-consumptive mechanism

In addition to shaping beaver foraging via direct killing, we suspect wolves, through their ambushing behaviour, constrain beaver foraging behaviour via non-consumptive mechanisms. Ambush predators are more likely to alter the behaviour of prey than cursorial predators because ambush predator cues are more concentrated in time and space [16]. As such, prey can more readily ascertain where predators wait in ambush and, in turn, adjust their behaviour to minimize encounters [72]. Through this process, ambush predators can substantially alter or reduce the movements and habitat domain of their prey through non-consumptive mechanisms (i.e. predation risk [4,16]). For example, sit-and-wait spiders reduced aspects of grasshopper habitat domain by up to 50–60% [73]. Even in the absence of predation, beavers have relatively narrow habitat domains given the energetic cost of cutting and transporting trees back to water [71]. However, wolf predation appears to narrow beaver habitat domain even further with wolves constraining the forest area predominantly used by beavers for foraging by up to 43–68% (figure 6). Teasing apart whether wolf predation as a consumptive or non-consumptive mechanism is most responsible for this pattern is difficult to determine. Yet, the answer is likely inconsequential because both forces operate simultaneously.

Ambush trails were longer than reference trails, indicating wolves chose to wait in ambush for beavers along longer trails. Beavers can be challenging prey for wolves to kill and are able to successfully evade wolves once attacked [42,54]. Distance to water is likely the most important factor influencing a beaver's probability of escape [23], and it is therefore not surprising that wolves wait in ambush on feeding trails where beavers are more available and vulnerable to predation. Previous work has demonstrated that wolves in the GVE choose ambush locations in response to the sensory abilities and anti-predator strategies of beavers [23]. We suspect wolves may select ambush locations based on the concentration or recency of beaver odorants, which are likely to be denser or fresher on longer trails because beavers spend more time there relative to shorter trails [25,74]. Wolves, like domestic dogs, have well-developed olfactory abilities that are almost certainly capable of detecting subtle differences in the recency and concentration of beaver odorants [75,76]. Other ambush predators, such as certain snake species, select ambush locations based on olfactory cues that confer prey availability or prey-rich patches [77–80]. Thus, wolves might wait in ambush along trails where beaver odorants are most concentrated because they assume this reflects increased beaver availability and their prospects of encountering beavers.

### (c) The indirect effects of predation on forests

Given the profound impacts of beaver foraging on forests (figure 1), we assert that if wolves constrain beaver foraging in any meaningful way—even if only by 1 or 2 m—then wolves indirectly influence the trajectory of forests around wetlands and shorelines. We have provided strong evidence that wolves do constrain foraging by disproportionately killing and ambushing beavers along longer feeding trails that are less numerous and used less frequently than shorter feeding trails (figures 2 and 5). In other words, wolves kill beavers at greater distances from water than would be expected based on the spatio-temporal availability of beavers when foraging. As such, wolves appear to limit the extent that beavers can disturb forests through foraging activities, thereby preventing beavers from altering the successional and ecological states of forests (figure 7). However, disturbance from beaver foraging affects many other ecological processes beyond forest structure and composition. Beaver foraging creates ecological heterogeneity and increases biodiversity around wetlands by increasing forest complexity—particularly by creating ‘messy forests’ with substantial dead and standing wood [71]. This in turn affects nutrient deposition and carbon storage, composition of lichen, bryophyte and plant communities, and habitat for, and abundance of, invertebrates, birds and mammals around beaver-altered environments [45,51,71]. Consequently, wolves, by reducing the amount of forest beavers can disturb, alter all of these ecological processes as well. In this sense, our work is analogous to previous work in the GVE which demonstrated that wolves indirectly alter all of the ecological processes associated with beaver-created wetlands by killing dispersing beavers and altering wetland creation-recolonization dynamics [46]. Combined, these studies indicate that wolves alter both riparian and terrestrial ecosystems by limiting and/or stopping the ecosystem engineering of beavers. Notably, the magnitude (forest altered per km^2^) of wolves' indirect effect on forests is almost certainly related to beaver colony density because beavers probably alter more forest per unit area at higher densities. Thus, as beaver density increases the indirect effect of wolves on forests likely increases as well.

Estimating or quantifying the extent to which wolves may indirectly alter forests by constraining beaver foraging behaviour is challenging. We suspect wolf predation primarily shifts the overall distribution of beaver foraging toward water (figure 6), though wolves may also influence the maximum distance from water beavers forage [37]. Put differently, beavers would forage more frequently and intensively at greater distances from water if wolves were not present. Researchers have surmised that predation constrains beaver foraging [23] based on differences in beaver foraging behaviour in predator-free and predator-dense environments. For example, non-native beavers in a largely predator-free system in Tierra del Fuego, South America foraged much farther from water than beavers in North American systems with higher predation pressure [38]. Beavers on islands with dense black bear populations in Apostle Island National Lakeshore, WI, USA appeared to forage substantially closer to water than beavers on nearby bear-free islands, where beavers foraged >200 m [36]. Similarly, beavers in Kostrama, Russia, where wolves and brown bears were abundant, foraged closer to water than beavers in a predator-free system in Germany [39]. Before wolves colonized Slate Island Provincial Park, Ontario, Canada beavers supposedly foraged >400 m from water [81]. Barnes & Mallik [37] noted that beavers in wolf-dense ecosystems typically foraged within 20 m of water—similar to beavers in the GVE (figure 2)—but beavers in wolf-free ecosystems foraged further from water. Based on beaver foraging patterns or other reasons, wolf predation was thought to limit beaver foraging distance near Chapleau, Ontario, Canada [37], Isle Royale National Park, USA [31] and Thunder Bay, Ontario, Canada [82]. Despite all of these observations, which were based almost entirely on patterns of beaver cut trees, no study has presented any predation-based data to illustrate how predation indirectly or directly constrains beaver foraging [23]. Our study presents direct evidence that predators do influence beaver foraging behaviour in a pattern that is consistent with many other studies across different systems.

In addition to altering forest structure and composition, wolves might also indirectly alter wetland dynamics in the GVE by reducing the area beavers predominantly forage in. Beaver-created wetlands undergo a recurring dynamic process of creation, occupation, abandonment and re-colonization [60,83]. One of the primary reasons beavers abandon wetlands is the depletion of food resources in close proximity to water [84,85]. At some point, beavers must decide to either forage further from water to secure more resources or abandon wetlands in search of more suitable habitats. Wolf predation, by constraining the distance beavers forage from water, effectively limits resource availability and in turn likely expedites resource depletion. Thus, wolves might indirectly influence and be connected to larger wetland dynamics by increasing rates of wetland abandonment and subsequent recolonization. We posit this is a logical conclusion if wolves constrain beaver foraging, yet we acknowledge that it is an untested hypothesis.

## 6. Conclusion

Central place foraging theory predicts foragers are at a higher risk of predation when foraging at greater distances from the central place [86]. Theoretical, experimental and indirect evidence supports this prediction, yet direct evidence has remained elusive. This evidence gap is in part due to the challenges of testing this hypothesis on natural predator–prey systems. We provide empirical evidence that a central place forager can be killed more frequently when foraging at greater distances from the central place, suggesting that predation is a significant ecological and evolutionary force shaping the movements and behaviour of central place foragers [29].

More importantly, we demonstrate how predators can have larger ecological effects by constraining the foraging behaviour of prey. Central place foragers can create ecological ‘halos’ by depleting food resources around the central place [26,31,87], which can have cascading ecological effects [51]. In some systems, these effects reverberate through food webs and alter ecological processes (e.g. nutrient deposition) and lower trophic levels [28,30]. Our work indicates predators can alter the typical distance that central place foragers will travel for resources, which ultimately influences the size of these ecological ‘halos’ [29]. Predators appear to limit the functional area available to central place foraging prey, and, in turn, limit the ecological effects generated by central place foraging prey.

## Data Availability

All data and code associated with this manuscript will be made publicly available via the Data Repository of the University of Minnesota at the following link: https://conservancy.umn.edu/handle/11299/253695 Additional information is provided in electronic supplementary material [88].
